# Energy driven mechanism of surrounding rock deformation and failure of mining roadway and classified control technology

**DOI:** 10.1038/s41598-025-98452-8

**Published:** 2025-04-30

**Authors:** Xuesheng Liu, Yu Zhang, Deyuan Fan, Yongqiang Zhao, Yudong Gao, Hongxi Pei, Zhihan Shi

**Affiliations:** 1 College of Energy and Mining Engineering, Shandong University of Science and Technology, Qingdao, 266590 China; 2 State Key Laboratory of Disaster Prevention and Ecology Protection in Open-pit Coal Mines, Shandong University of Science and Technology, Qingdao, 266590 China; 3 National Energy Group Ningxia Coal Industry Co, Ltd., Yinchuan, 750011 China

**Keywords:** Mining roadway, Energy-driven, Large deformation, Impact instability, Classified control, Civil engineering, Coal

## Abstract

The energy evolution of surrounding rock under mining influence has a crucial impact on its stability. Taking the I0104_3_02 transportation roadway of Shuangma as the engineering background, the energy evolution law of surrounding rock in mining roadway was obtained by FLAC3D. The energy influence range and value of elastic energy and dissipated energy at both sides of the roadway gradually increase with the mining. The energy value at the face end reaches its maximum, with the dissipated energy release area forming a butterfly-shaped distribution and the elastic energy accumulation area forming a trapezoidal accumulation pattern. Subsequently, the surrounding rock of the roadway is divided into energy consumption zone, energy supply zone, and unaffected zone based on the range of two energies. Then, the types of surrounding rock deformation and failure are identified from two aspects: energy level K and energy release rate V. When K ≥ 1 and V ≥ 1, the surrounding rock of the roadway undergoes impact instability; when V < 1, the surrounding rock of the roadway undergoes large deformation failure. Furthermore, "Multi-Level pressure yielding" and "Relief-Support" control technology are proposed for the two types of failure, and field verification has been carried out in Shuangma and Xinjulong.

## Introduction

During the process of underground mineral resource mining, the stress field around the ore body and the filling body will be redistributed, forming a mining stress field^1^. With the progress of mining activities, the roadway is not only affected by the original rock stress but also by the mining stress^2^. The complexity, particularity, and variability of the stress environment of the surrounding rock in mining roadway make the deformation and failure of the roadway complex and highly variable.

The deformation and failure of surrounding rock in roadway are considered to be caused by the plastic zones generated by the surrounding rock^3^. Hermann Kastner^4^ proposed the classic plastic zone theory of surrounding rock in roadway, which laid the theoretical foundation for the study of the failure of surrounding rock in roadway under equal pressure stress fields. Wang et al.^5^ derived the approximate solution of the boundary equation of the plastic zone of surrounding rock in non-equal pressure circular roadway considering the effect of support force, and obtained the influence of support resistance on the range and shape of the plastic zone of surrounding rock in roadway. Guo et al.^6^ theoretically derived the boundary equation of the plastic zone of surrounding rock in circular roadway under non-equal pressure stress conditions and discovered the "butterfly-shaped" irregularity of the plastic zone of surrounding rock in roadway. Hong et al.^7^ studied and found that high-level stress has the greatest impact on the failure of deep roadway. Under high-level stress, the sides of the roadway are severely squeezed, the convergence of both sides increases, the roof and floor are severely deformed, and there is a high possibility of rupture at the arch. Shang et al.^8^ used numerical simulation analysis to determine the cause of roadway instability when the roof rock mass has high strength and the floor has low strength, and summarized the characteristics of deep roadway instability.

In recent years, many experts at home and abroad have conducted in-depth research on the stability of surrounding rock in mining roadway and proposed effective support design schemes. Jing^9^ used the "anchor-frame" joint support technology to control the soft and broken roadway, established the "anchor-frame" joint support composite arch model, and calculated the ultimate bearing capacity of this type of structure. Qing et al.^10^ starting from the perspective of the surrounding rock’s existence of loose and broken zones, deeply analyzed the types of roadway support loads, and based on a large number of research results, established the "loose circle support theory". Daemen^11^ conducted research on the stability of deep-buried high-stress soft rock roadway, considering the effect of the self-weight of the rock mass in the plastic zone, and established a functional relationship between the surrounding rock and support characteristic curves. Zhang et al.^12^ used experimental and numerical simulation methods to compare and analyze the stability of soft rock roadway under the action of steel arch frames and grid steel arch frames, and proposed a reasonable support plan for controlling the large deformation of soft rock. Zhao et al.^13^ analyzed the law of change in the interaction force between roadway surrounding rock and support, and the research results showed that compared with grid steel arch support, the surrounding rock pressure increased by about 80% under the condition of steel arch support.

At present, domestic and foreign scholars generally judge the instability mechanism of failure from the classical elastoplastic strength criteria and analyze the distribution range of surrounding rock failure and targeted support optimization design from the perspective of stress.^14^ These research works have a certain reference for guiding the mechanism of deformation and failure of surrounding rock in mining roadway and the stability of surrounding rock in roadway. However, there is not enough in-depth discussion on the energy-driven mechanism of surrounding rock deformation and failure from the perspective of energy, and no classification control technology for surrounding rock deformation and failure has been proposed. Therefore, based on the transportation roadway of the I0104_3_02 working face in Shuangma Mine as the engineering background, this paper uses a combination of numerical simulation and field practice to study the energy partition evolution law of surrounding rock in mining roadway, reveal the mechanism of deformation and failure of surrounding rock in mining roadway, construct a type identification of surrounding rock deformation and failure, and propose corresponding surrounding rock deformation control technology, which is verified in combination with engineering practice. The research results are of great practical significance for ensuring the stability of surrounding rock in mining roadway.

## Mechanism of deformation and failure of surrounding rock in mining roadway

### Law of energy evolution of surrounding rock in mining

#### Site engineering conditions

The I0104_3_02 working face of Shuangma Mine has an inclined length of 266.5 m, a strike length of 1241.4 m, and a burial depth of about 300 m. The coal seam structure is simple, with an average coal seam thickness of about 1.71 m, no intercalations, and belongs to a stable coal seam. The roof of the coal seam is mainly sandstone, with an average thickness of 10.91 m, and the floor is mainly siltstone, with an average thickness of 15.75 m. The physical and mechanical parameters of the coal seam and rock layers are shown in Table 1.

**Table 1:** The physical and mechanical parameters.

Lithology	Density (g/cm^3^)	Compressive strength (MPa)	Tensile strength (MPa)	Elastic modulus (GPa)	Poisson ratio	Friction (°)	Cohesion (MPa)
Siltstone	2.6	35.43	4.22	2.78	0.21	37.69	1.08
Fine-grained sandstone	2.2	32.89	2.02	8.92	0.33	63	3.31
Coal	1.3	17.34	1.97	1.34	0.25	32.15	0.34
Siltstone	2.7	35.43	4.22	2.78	0.21	37.69	1.08
Medium-grained sandstone	2.4	58.32	4.22	5.79	0.25	41.51	4.97
Fine-grained sandstone	2.2	32.89	2.02	8.92	0.33	63	3.31

Due to the strong mining pressure manifestations during the mining period, the surrounding rock deformation and failure are abnormal, which seriously affects the normal production of the working face, and the cost and difficulty of repair are high. To prevent this problem from occurring again, it is planned to carry out related research on the transportation roadway of the I0104_3_02 working face.

#### Numerical simulation scheme

**Fig. 1:**
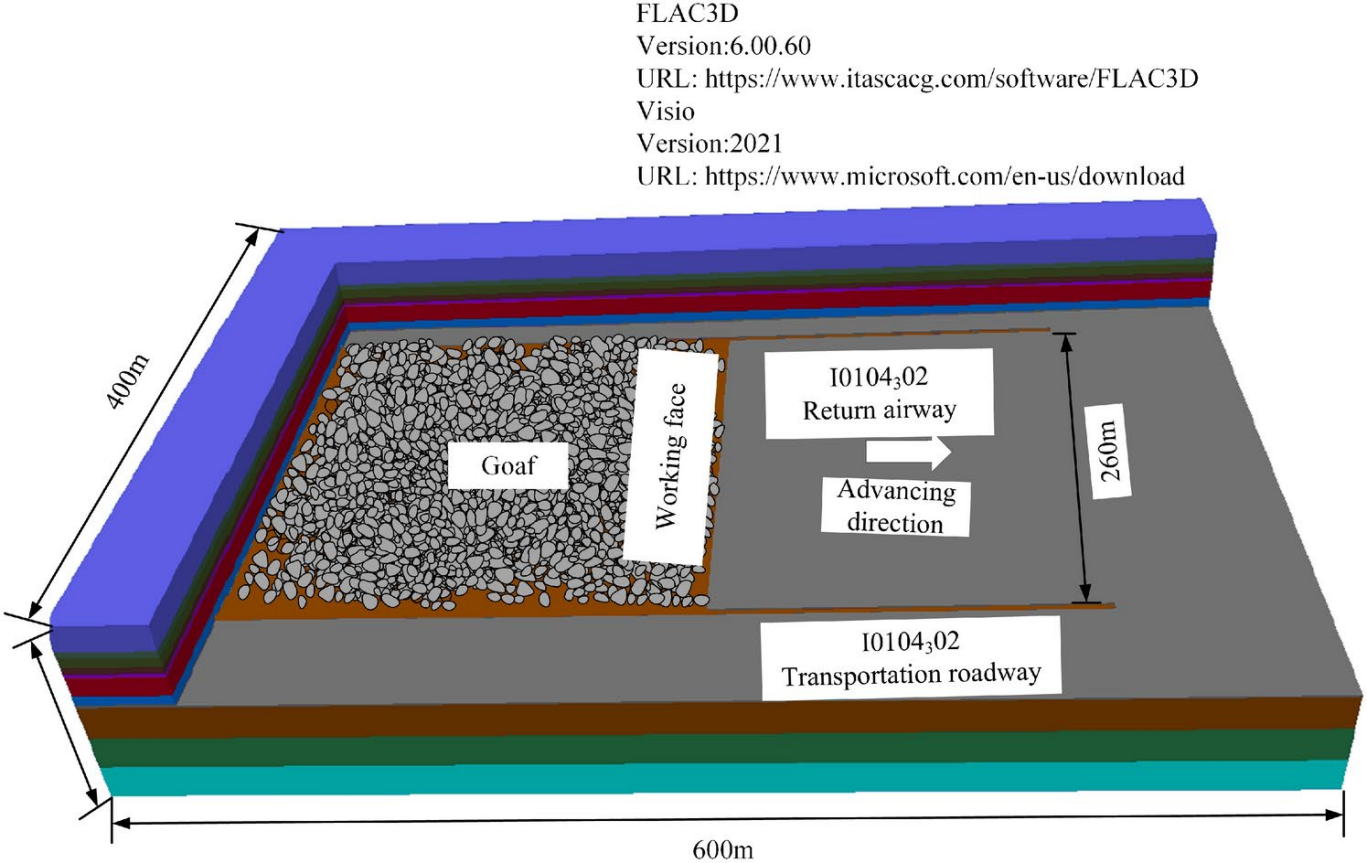
Numerical simulation model.

Based on the actual engineering geological conditions, the FLAC3D numerical simulation software is used to establish a numerical model. The length, width and height of the model are 600 m, 400 m and100 m, respectively. In the model, the width and height of working face are 260 m and 2 m, respectively, and those of roadway are both 4 m, as shown in Fig. 1. The model is a free surface on top, with a vertical load of 7.5 × 10^6^ kN applied, and displacement constraints are applied on all sides and bottom, using the Mohr–Coulomb constitutive model.

**Fig. 2:**
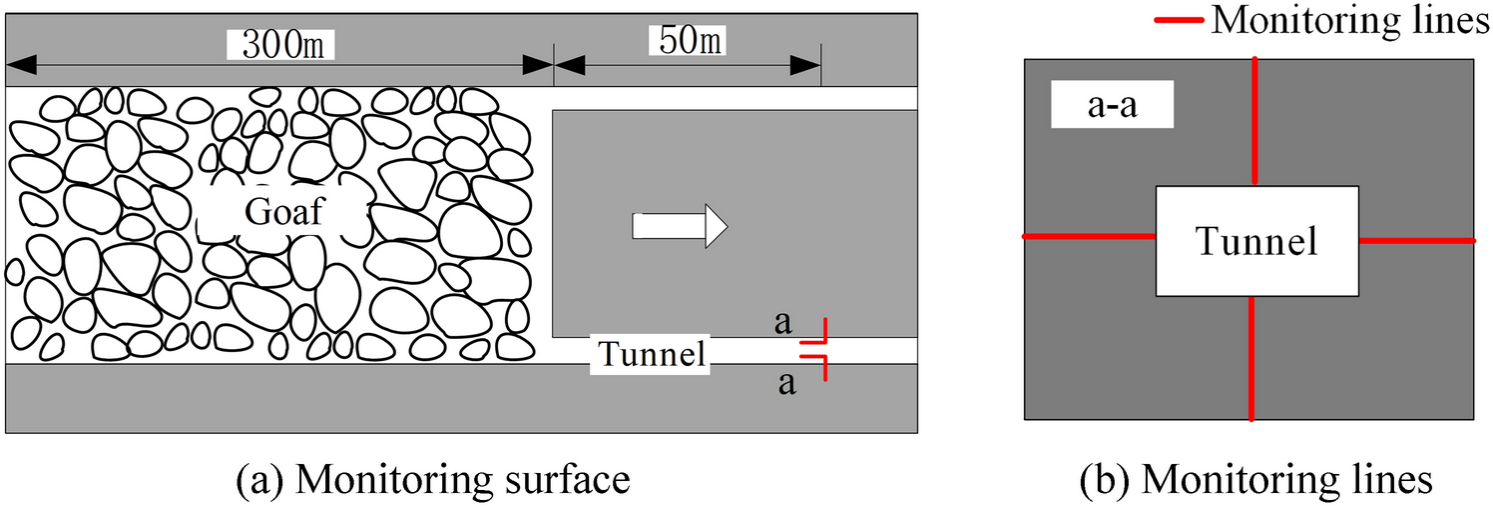
Monitoring surface and monitoring lines layout.

To explore the impact of mining activities on the energy changes of surrounding rock in roadway at different distances from the working face, the energy of the roof, floor, both sides, and floor rock units of the surrounding rock is monitored. A measuring line is arranged 350 m away from the working face. To eliminate the influence of boundary effects and insufficient mining on the monitoring results, monitoring is started after the working face has retreated 300m, and then a record is made every 10m the working face retreats, with a total of 6 groups monitored. The layout of the monitoring surface measuring line is shown in Fig. 2.

#### Simulation results analysis

**Fig. 3:**
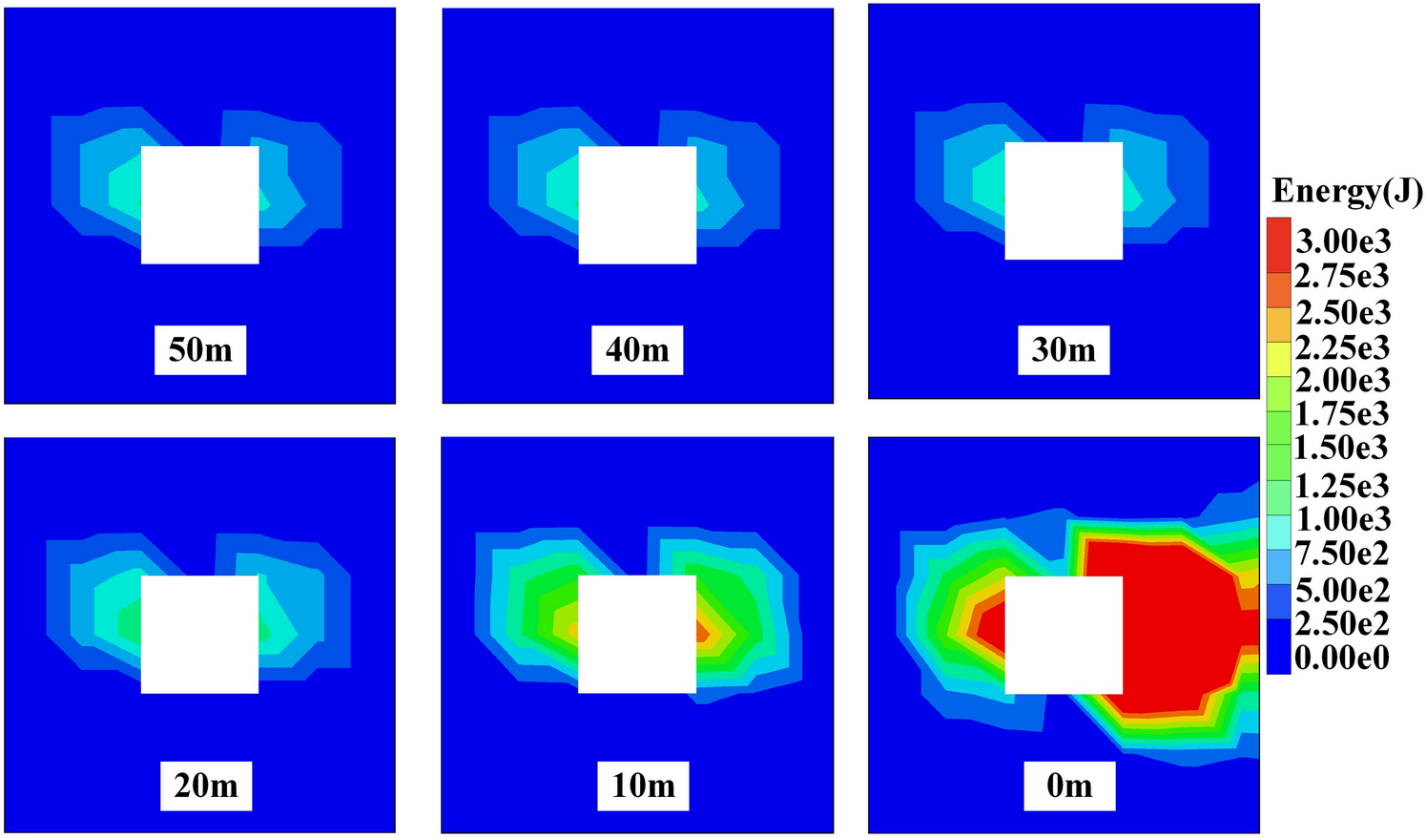
Dissipated energy distribution of surrounding rock.

Before mining, the surrounding rock mass of the roadway is in a state of stress balance. Mining activities will disturb the original relatively balanced geostress field, causing the stress field of the surrounding rock to redistribute and produce stress concentration.^15^ The energy field is also like this. The surrounding rock mass is the carrier of energy. When the coal seam is excavated, due to the influence of mining, energy is transmitted and accumulated towards the edge of the roadway. If the elastic energy accumulated in some areas of the surrounding rock exceeds the limit it can bear, it will lead to a surge in dissipated energy of the surrounding rock of the roadway, resulting in plastic deformation or failure.^16^ The energy cloud map of the surrounding rock of the roadway is shown in Figs. 3 and 5.

When the working face advances to 30–50 m from the measuring line, the dissipated energy distribution of the surrounding rock of the roadway is mainly butterfly-shaped, symmetrically distributed with the centerline of the roadway as the axis of symmetry, and dissipated energy concentration appears on both sides of the roadway. At this time, the dissipated energy release mainly comes from the stress redistribution after the roadway excavation, and is less affected by mining. When the working face advances to 20 m from the measuring line, the dissipated energy of the surrounding rock of the roadway begins to be affected by mining, and the range of dissipated energy distribution spreads outward. When the working face advances to 10 m from the measuring line, the range of dissipated energy distribution continues to spread outward, and the dissipated energy on both sides of the roadway is significantly increased compared to when the working face advances to 20 m from the measuring line. When the working face advances to the measuring line, the dissipated energy of the surrounding rock of the roadway is no longer symmetrically distributed, and dissipated energy is released at the roof and floor. The dissipated energy release is most affected by mining, and the dissipated energy released by both sides of the roadway reaches its maximum value at this time.^17^

**Fig. 4:**
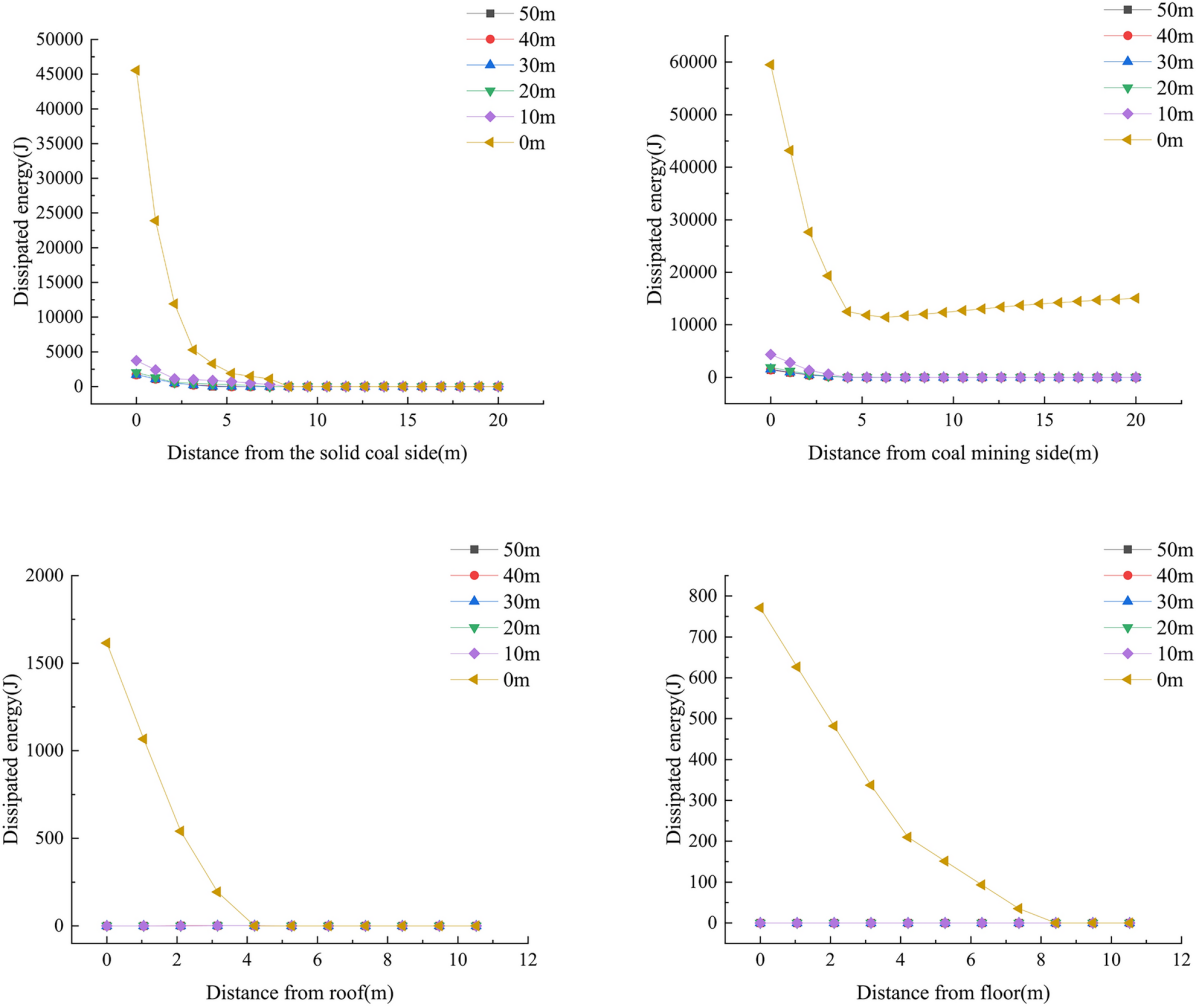
Curve of dissipated energy distribution.

In order to explore the impact of the working face advancing to different distances on the range of energy distribution, the dissipated energy monitoring curve of the surrounding rock of the roadway is extracted. The dissipated energy distribution curve of the surrounding rock of the roadway at different distances from the working face is shown in Figs. 4 and 6.

**Fig. 5:**
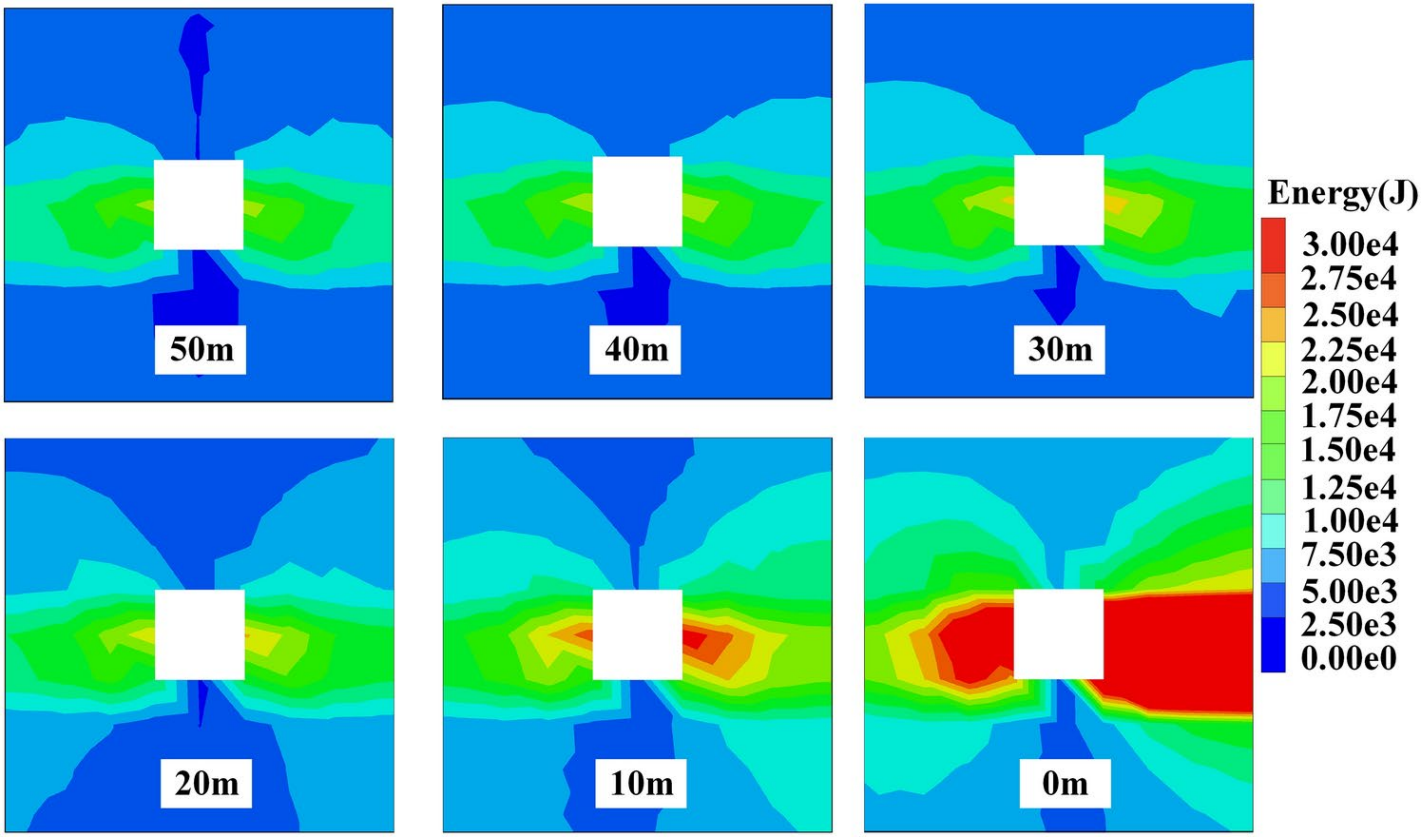
Elastic energy distribution of surrounding rock.

According to Fig. 4, when the working face advances to between 30 and 50 m from the measuring line, the dissipated energy distribution boundary on both sides of the roadway is 4 m, and there is no distribution of dissipated energy at the roof and floor. As the working face continues to advance, the range of dissipated energy distribution also gradually expands. When the working face advances to 10 m from the measuring line, the distribution range of dissipated energy on both sides reaches the maximum, of which the solid coal side is about 10m, and the mining side is about 12 m. When the working face advances to the measuring line, the size of dissipated energy on both sides continues to increase, the range of dissipated energy distribution spreads towards the roof and floor of the roadway, and the distribution boundary distances are 4 m and 6 m, respectively.

When the working face advances to between 30 and 50 m from the measuring line, the distribution of elastic energy of the surrounding rock of the roadway is mainly trapezoidal, symmetrically distributed with the centerline of the roadway as the axis of symmetry. At this time, the accumulation of elastic energy is still the result of stress redistribution, and is less affected by mining. When the working face advances to 20 m from the measuring line, the elastic energy of the surrounding rock of the roadway begins to be affected by mining, and the range of elastic energy distribution spreads outward. When the working face advances to 10m from the measuring line, the range of elastic energy distribution continues to spread outward, and the elastic energy on both sides of the roadway is significantly increased compared to when the working face advances to 20 m from the measuring line. When the working face advances to the measuring line, the elastic energy of the surrounding rock of the roadway is no longer symmetrically distributed, and elastic energy accumulates at the roof and floor. The accumulation of elastic energy is most affected by mining, and the amount of elastic energy released at the mining side is significantly greater than that at the solid coal side, and the elastic energy accumulated on both sides of the roadway reaches its maximum value at this time.

**Fig. 6:**
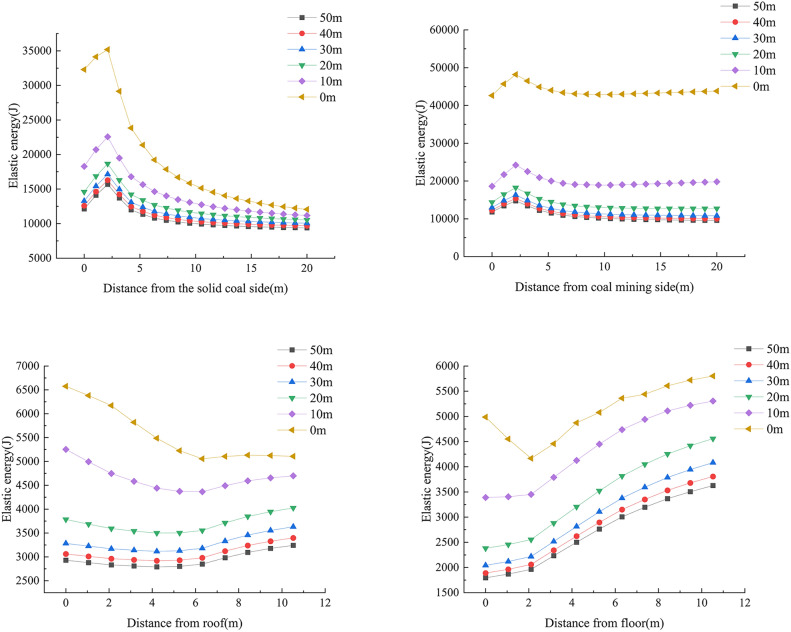
Curve of elastic energy distribution.

According to Fig. 6, when the working face advances to between 30 and 50 m from the measuring line, the distribution boundary of elastic energy on both sides of the roadway is 6 m, and there is no distribution of elastic energy at the roof and floor. As the working face continues to advance, the range of elastic energy distribution also gradually expands. When the working face advances to 10m from the measuring line, the distribution range of elastic energy on both sides reaches the maximum, of which the solid coal side is about 19 m, and the mining side is about 20 m. When the working face advances to the measuring line, the size of elastic energy on both sides continues to increase, the range of elastic energy distribution spreads towards the roof and floor of the roadway, and the distribution boundary distances are 5 m and 9 m, respectively.

From the numerical simulation, it can be known that the advance influence range of the I0104_3_02 working face is 30 m. Within the advance influence range, the energy influence range and value at both sides of the roadway gradually increase with the advancement of the working face, and reach the limit at 10m in advance. The energy influence area spreads from both sides of the roadway towards the roof and floor. The energy value at the head of the working face reaches its maximum, with the dissipated energy release area forming a butterfly-shaped distribution, and the elastic energy accumulation area forming a trapezoidal accumulation pattern, and the range of elastic energy accumulation is greater than the range of dissipated energy release.

### Surrounding rock partition model

Research has shown that the accumulation and release of energy are the root causes of deformation and failure of surrounding rock in roadway.^18^ Therefore, when the surrounding rock of the roadway deforms and destroys, there must be some rock mass that consumes energy, some rock mass that supplies energy, and some rock mass that is far from the working face and is not affected by mining activities.

**Fig. 7:**
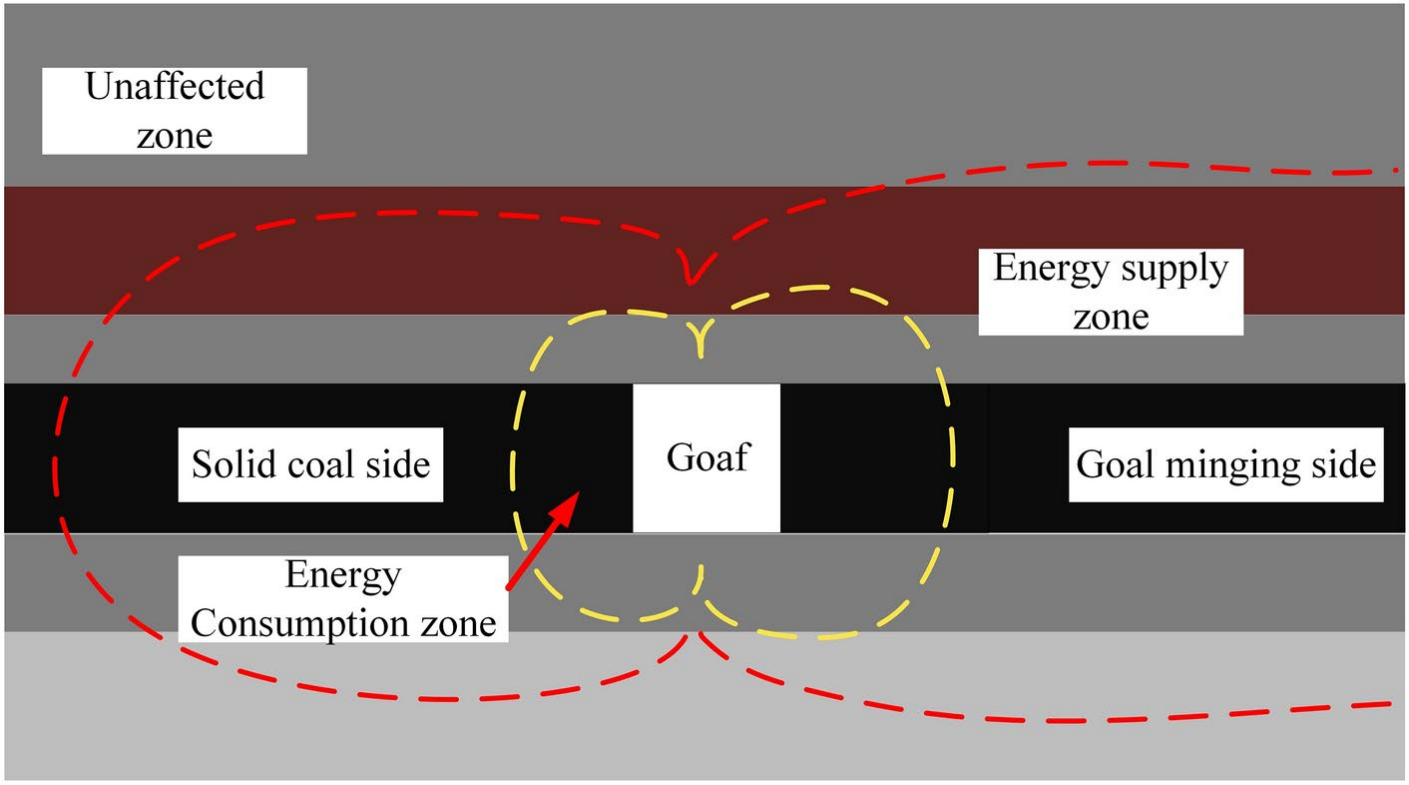
Schematic diagram of energy partition of surrounding rock.

Based on the results of numerical simulation, the surrounding rock of the roadway is divided into three areas based on the boundary of the dissipated energy release range and the boundary of the elastic energy accumulation range. This paper will call them the energy consumption zone, the energy supply zone, and the unaffected zone, respectively. The division of energy zones of surrounding rock is shown in Fig. 7.

The area close to the working face is greatly affected by mining, and the coal mass in the area is affected by both the original rock stress and the mining stress. The energy accumulated by the surrounding rock of the roadway is in a state of release at this time, which is the energy consumption zone. The area slightly farther from the working face is still affected by mining, but its elastic energy accumulation is much greater than the release of dissipated energy. In terms of structure, it is not destroyed, and in terms of energy, it is the elastic energy transmitted to the energy consumption zone, which is the energy supply zone. The coal mass far from the working face is only affected by the original rock stress. The pressure in the area is small, and during the mining period, it is still in the elastic state in the stress–strain curve, which is the unaffected zone.

#### Identification of deformation and failure types

The causes of deformation and failure of surrounding rock in roadway are complex, there are many types of failure, and the hazards are great. In actual production, there is a lack of identification of deformation and failure of surrounding rock in roadway, which leads to a single type of roadway support, especially when facing impact instability and large deformation failure, it cannot ensure the stability of surrounding rock in roadway. Therefore, the identification of deformation and failure types of surrounding rock in roadway is of certain help to the stability of surrounding rock in mining roadway and the safe and efficient production of mining areas. This paper proposes the identification of deformation and failure types of surrounding rock in roadway from the perspective of energy level and energy release rate.

It is known from the stress–strain curve of rocks that when the external energy input to the rock reaches the peak energy threshold during the loading process of rocks, it enters the failure and instability stage.

The peak value energy threshold (*U*_*s*_) of a single rock standard specimen during indoor experiments can be expressed as:

1 $$U_{s} = \int_{0}^{{\varepsilon_{s} }} \sigma d\varepsilon$$

The energy (*U*_*t*_) accumulated by the in-situ rock layer at any time t_1_ − t_2_ can be expressed as:

2 $$U_{t} = \int_{{\varepsilon_{1} }}^{{\varepsilon_{2} }} {\int_{{t_{1} }}^{{t_{2} }} {\sigma_{t} } d\varepsilon } dt$$

The energy (*U*_*0*_) accumulated by the original rock stress can be expressed as:

3 $$U_{0} = \frac{{\sigma_{0}^{2} }}{2E}$$

The energy (*U*_*d*_) generated due to different dynamic loads during the mining process can be expressed as:

4 $$U_{{\text{d}}} = \sum\limits_{i = 1}^{n} {U_{i} X_{i}^{{ - \alpha_{i} }} }$$

The ratio of the total energy value that the rock layer currently bears to the peak energy threshold is defined as the energy level identification coefficient *K*. When *K* ≥ 1, it indicates that the total energy value currently borne by the rock layer has reached or exceeded the peak energy threshold, and the roadway will become unstable. When *K* < 1, it indicates that the total energy value currently borne by the rock layer has not reached the peak energy threshold, and the roadway will not become unstable.

5 $$K = \frac{{\int_{{\varepsilon_{1} }}^{{\varepsilon_{2} }} {\int_{{t_{1} }}^{{t_{2} }} {\sigma_{t} } d\varepsilon } dt + \frac{{\sigma_{0}^{2} }}{2E} + \sum\limits_{i = 1}^{n} {U_{i} X_{i}^{{ - \alpha_{i} }} } }}{{\int_{0}^{{\varepsilon_{s} }} \sigma d\varepsilon }}$$

where $\varepsilon_{{\text{s}}}$ is the strain corresponding to the failure peak stress; $\sigma_{t}$ is the stress at time *t*; $\sigma_{0}$​ is the original rock stress; *E* is the elastic modulus; *U*_i_ is the energy released by different dynamic loads at the source; *X*_i_ is the distance of different sources from the roadway; and $\alpha_{i}$​ is the energy propagation attenuation index of different dynamic loads in the coal-rock medium.

**Fig. 8:**
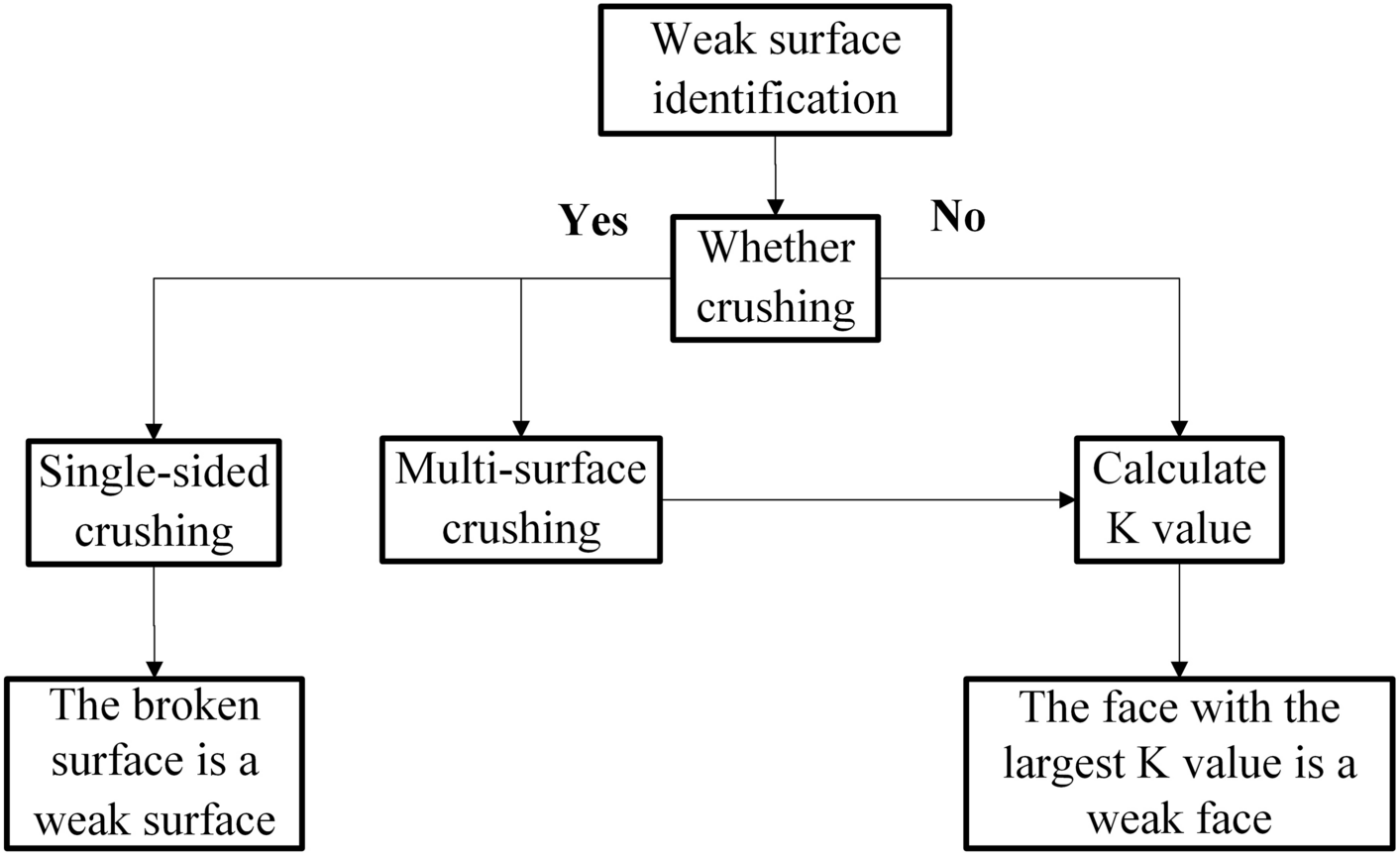
Flowchart for weak face identification.

When the roadway undergoes deformation and failure, the elastic energy accumulated in the surrounding rock of the roadway is released and propagated to the weak face, which first fails, leading to the overall instability of the mining roadway. Therefore, it is necessary to identify the weak face in advance for the control of mining roadway.^18^ The peak energy threshold of the surrounding rock of the roadway will not change due to the external environment before failure. From Eq. (5), it can be concluded that the larger the *K* value, the greater the energy currently borne by the surrounding rock of the roadway, the lower the ability of the surrounding rock of the roadway to resist instability risk, and the more likely it is to become unstable. Therefore, *K* can be used to identify the weak faces of deformation and failure, and the identification process is shown in Fig. 8.

Roadway deformation and failure are manifestations of the magnitude and release rate of energy on weak face within the roadway. For the surrounding rock mass of the roadway, the type of failure can be identified by the evolution of energy during the failure process. By analyzing the relationship between the total energy, elastic strain energy, and the dissipated energy required for plastic deformation based on the complete stress–strain curve of rocks, the release rate of accumulated elastic energy is taken as one of the criteria for identifying the type of deformation and failure.^19^

**Fig. 9:**
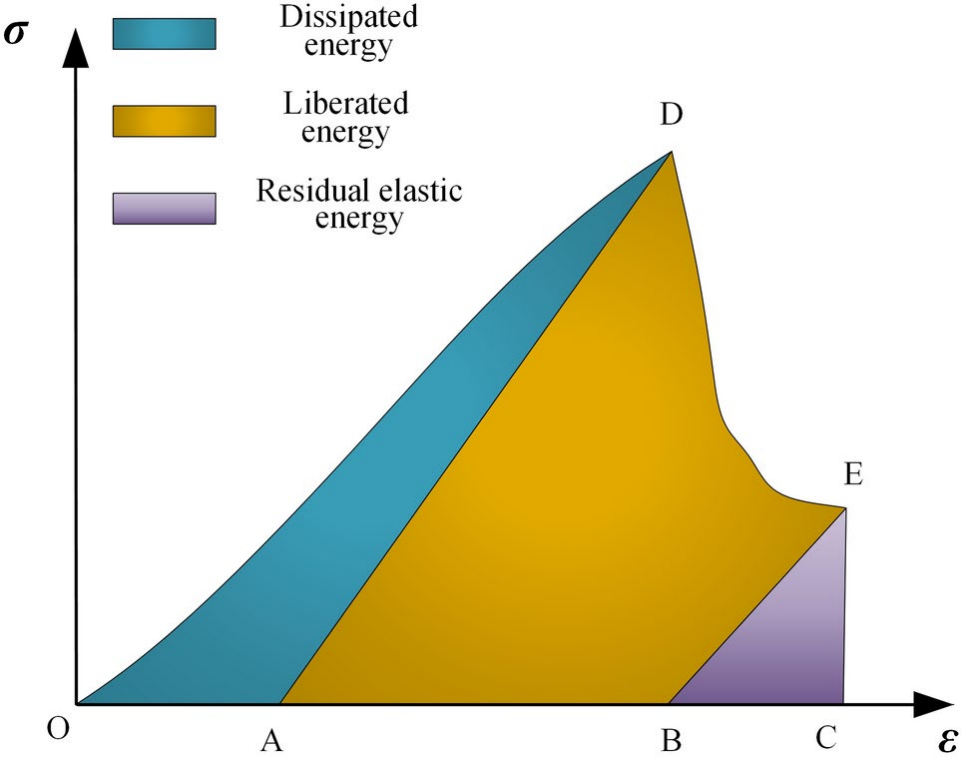
The energy evolution characteristics of pre-peak and post-peak stages.^20^

As shown in Fig. 9, area *S*_*ODA*_ represents the dissipated energy required for fracturing and plastic deformation, area *S*_*ABD*_ represents the elastic energy stored during the loading process. Area *S*_*ABED*_ is the energy released when the coal-rock fails, and area *S*_*BCE*_ is the residual elastic energy after failure.

6 $$S_{ABD} = \frac{{\sigma_{{\text{s}}}^{2} }}{2E}$$

7 $$S_{BCE} = \frac{{\sigma_{r}^{2} }}{{2E_{r} }}$$

8 $$S_{BCED} = \int_{{\varepsilon_{D} }}^{{\varepsilon_{E} }} \sigma d\varepsilon$$

9 $$S_{ABED} = \frac{{\sigma_{{\text{s}}}^{2} }}{2E} + \int_{\varepsilon d}^{{\varepsilon_{r} }} \sigma d\varepsilon - \frac{{\sigma_{r}^{2} }}{{2E_{r} }} = \frac{{2EE_{r} \int_{{\varepsilon_{D} }}^{{\varepsilon_{r} }} \sigma d\varepsilon + E_{r} \sigma_{{\text{s}}}^{2} - E\sigma_{r}^{2} }}{{2EE_{r} }}$$

where $\sigma_{r}^{{}}$ is the residual stress; $E_{r}$ is the elastic modulus after failure; $\varepsilon_{E}^{{}}$​ is the strain at point *E*; $\varepsilon_{D}^{{}}$​ is the strain at point *D*.

The rate of energy release when the surrounding rock of the roadway undergoes deformation and failure is *V*_1_.

10 $$V_{1} = \frac{{2EE_{r} \int_{{\varepsilon_{D} }}^{{\varepsilon_{r} }} \sigma d\varepsilon + E_{r} \sigma_{{\text{s}}}^{2} - E\sigma_{r}^{2} }}{{2\Delta T_{1} EE_{r} }}$$

where $\Delta T_{1}$ is the time at which deformation and failure occur in the surrounding rock of the roadway.

Assuming that the release speed of the peak energy threshold of the roadway instability is *V*_2_.

11 $$V_{2} = \frac{{\int_{0}^{{\varepsilon_{s} }} \sigma d\varepsilon }}{{\Delta T_{2} }}$$

where $\Delta T_{2}$ is the release time of the peak energy threshold of the roadway instability.

12 $$V = \frac{{2EE_{r} \Delta T_{2} \int_{{\varepsilon_{D} }}^{{\varepsilon_{r} }} \sigma d\varepsilon + \Delta T_{2} E_{r} \sigma_{{\text{s}}}^{2} - \Delta T_{2} E\sigma_{r}^{2} }}{{2\Delta T_{1} EE_{r} \int_{0}^{{\varepsilon_{s} }} \sigma d\varepsilon }}$$

**Fig. 10:**
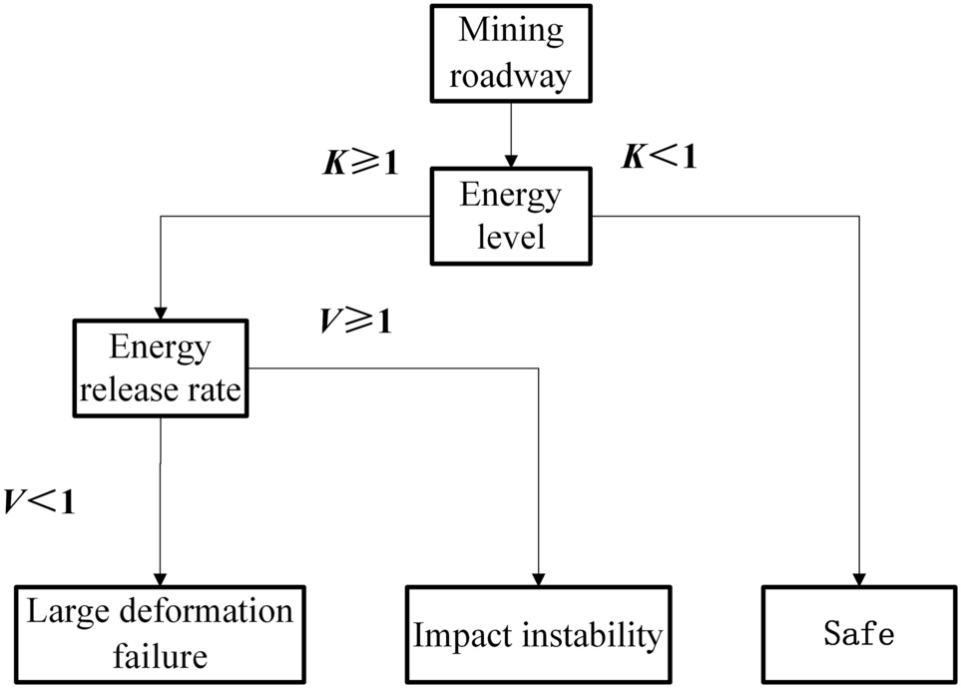
Flowchart for deformation and failure type identification.

The ratio of the rate of energy release when the surrounding rock of the roadway undergoes deformation and failure to the release speed of the peak energy threshold of roadway instability is defined as the rate identification coefficient *V*. If *V* ≥ 1, impact instability occurs; if *V* < 1, large deformation failure occurs. The identification process is shown in Fig. 10.

### Classified control technology

#### "Multi-Level pressure yielding" control technology

Traditional rigid support controls the large deformation of roadway by improving the stiffness and strength of the support structure.^21,22^ Although the rigid support scheme can suppress the deformation of the surrounding rock to a certain extent, when encountering soft surrounding rock, the force on the support system continues to increase, leading to harmful loosening and excessive displacement of the surrounding rock. With the continuous increase of surrounding rock deformation, the plastic zone continues to increase, eventually leading to the collapse of the roadway and causing huge losses.^23^

Pressure yielding support is based on traditional rigid support, by adding some pressure yielding structures, allowing the surrounding rock to deform to a certain extent to release ground stress and reduce the pressure on the support structure, thus achieving stable control of the surrounding rock. Pressure yielding support only sets Pressure yielding structures at the end, and the pressure yielding amount is relatively small, so the control effect on the surrounding rock of large deformation roadway is still limited.

**Fig. 11:**
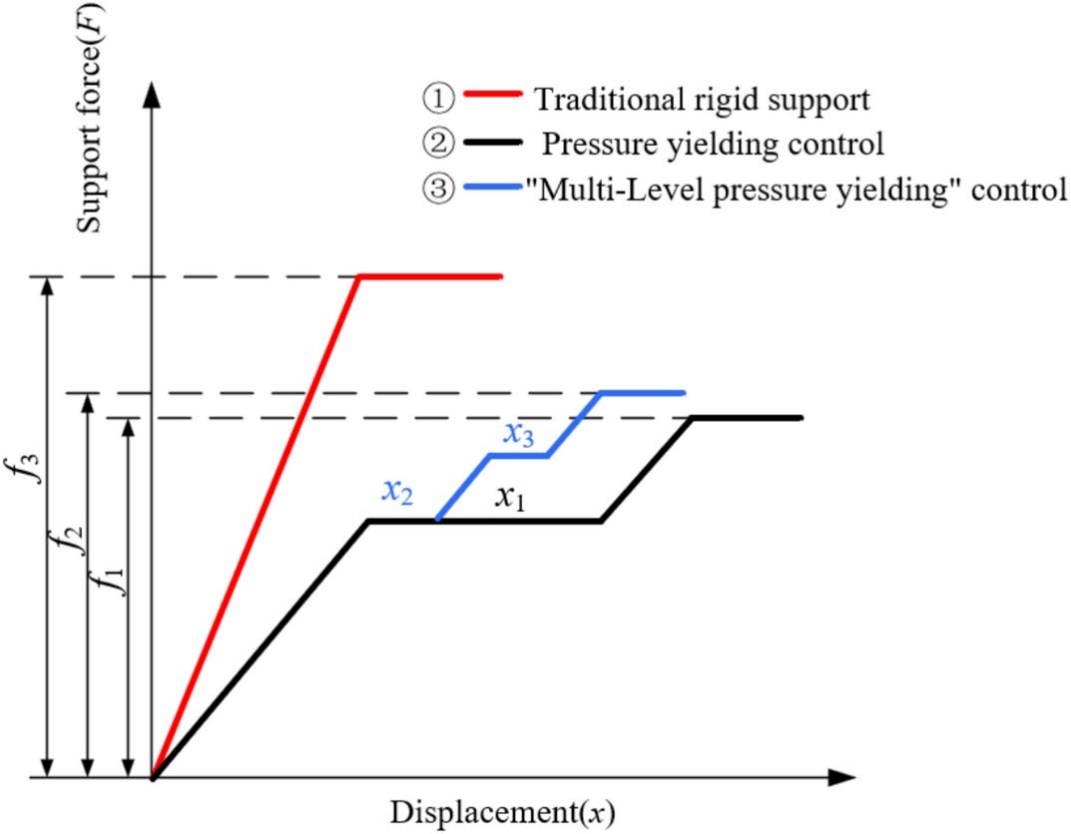
The force characteristics of structure.

Therefore, aiming at the problem that traditional rigid support and pressure yielding support cannot well solve the control of surrounding rock of roadway at risk of large deformation, the "multi-level pressure yielding" control technology for surrounding rock of roadway at risk of large deformation is proposed.^24^ Multi-level Pressure yielding support is further developed based on pressure yielding support. It can more finely control the deformation of the surrounding rock by setting multiple pressure yielding levels, adapting to the deformation needs of different stages and degrees. The force characteristics of surrounding rock and structure under different support measures are shown in Fig. 11. Curve ① is traditional rigid support, and the support force *f*_3_ bears is obviously higher than curves ② and ③. The force borne by the support structure is large, and stress concentration areas are prone to appear, which is not suitable for surrounding rock of roadway at risk of large deformation. Curve ② is pressure yielding support, which adds pressure yielding anchors and other pressure yielding structures based on traditional rigid support. By allowing a certain amount of displacement of the surrounding rock of the roadway, it has the ability to deform together with the surrounding rock, thus achieving the effect of releasing the stress of the surrounding rock. The support force it withstands is denoted as *f*_1_, and the yielding displacement is denoted as *x*_1._ Curve ③ is multi-level pressure yielding support, which continues to add pressure yielding components based on pressure yielding support, so that the pressure yielding amount of pressure yielding support is released in stages. The support force it withstands is denoted as *f*_2._ Its pressure yielding amount is the same as pressure yielding support, which is (x_3_ + x_4_). Comparing curves ② and ③, curve ③ releases the total pressure yielding amount of curve ② by increasing the number of pressure yielding times, allowing it to intersect with the characteristic curve of the surrounding rock earlier and enter a stable state earlier. In summary, multi-level pressure yielding support bears less support resistance than rigid support, and the deformation of surrounding rock is reduced compared to pressure yielding roadway, and the surrounding rock enters a stable state earlier.^25^ Multi-Level pressure yielding support can effectively control the surrounding rock of roadway at risk of large deformation.

### "Relief -Support" control technology

It is known that when impact instability occurs, the coal mass will be instantly thrown out. As the surrounding rock energy continues to accumulate and release, it leads to the failure of the roadway sides from the surface to the inside. On the one hand, it is necessary to cut off the stress transmission path by relief, transferring the peak value of support pressure to a deeper part.^26^ On the other hand, it is necessary to reinforce the shallow part of the roadway by strengthening support to prevent the broken coal mass in this area from being thrown out and causing impact instability.^27^

**Fig. 12:**
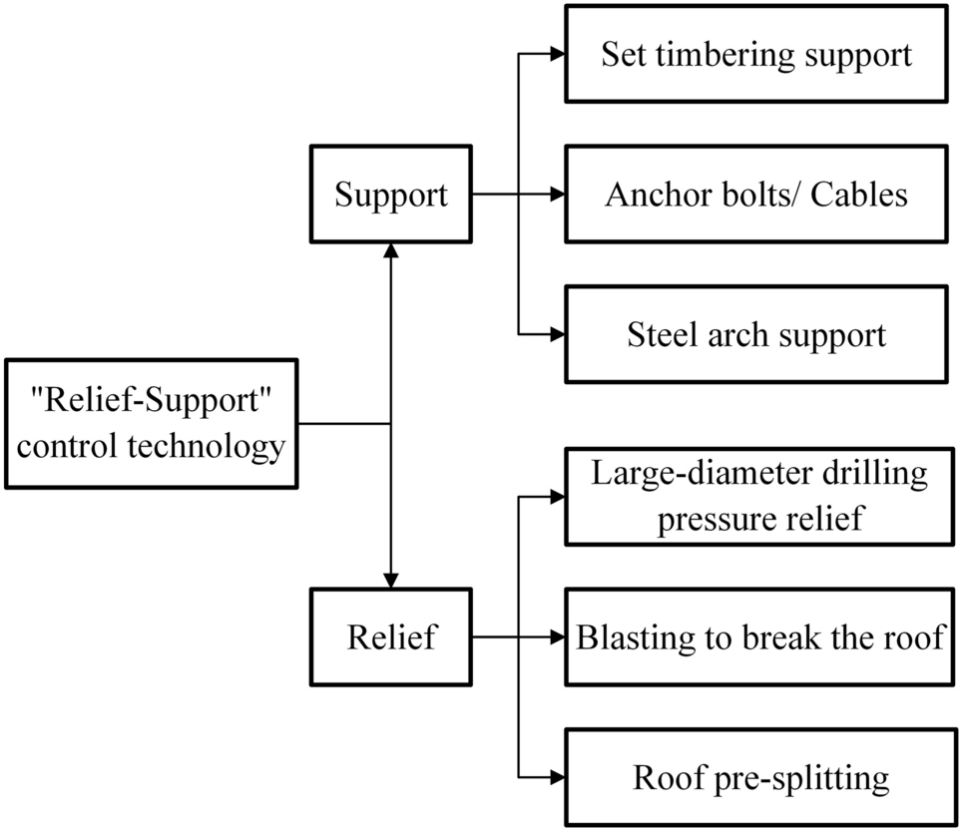
Schematic diagram of "Relief-Support" control technology.

**Fig. 13:**
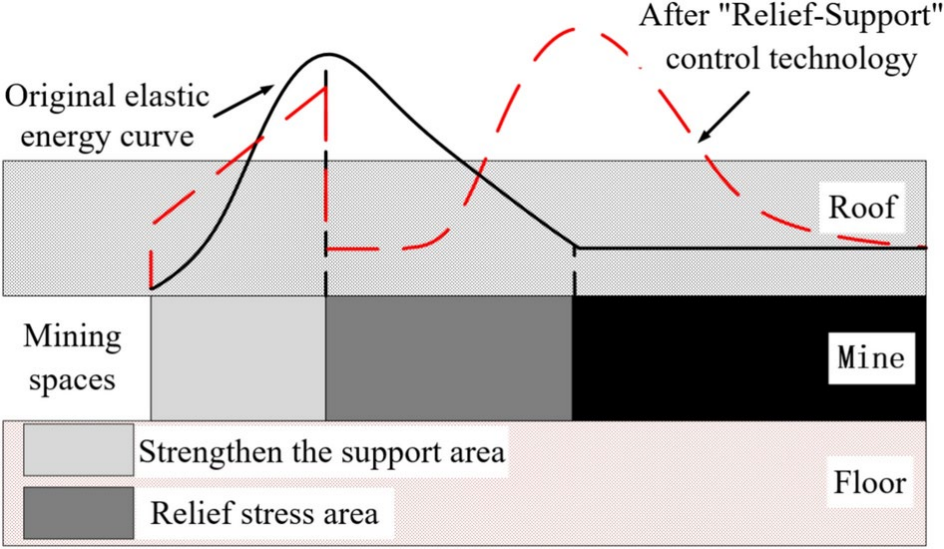
Schematic diagram of "Relief-Support" control technology.

Based on this, the "Relief-Support" control technology for surrounding rock of roadway at risk of impact instability is proposed, as shown in Fig. 12. On the one hand, technologies such as anchor bolts/cables, and grouting are used to support the energy release area of the coal roadway sides, reinforcing the broken area into a structure that can withstand pressure. On the other hand, technologies such as blasting to break the roof, large-diameter drilling pressure relief, and roof pre-splitting are used to relief the coal mass or roof and floor, transferring the high-pressure peak area or energy accumulation area to the deep part of the sides, and keeping the high-energy accumulation area away from the mining space.^28^ The "Relief-Support" control technology is shown in Fig. 13.

## Engineering case studies

### ***Transportation roadway of I0104***_***3***_***02 working face in Shuangma mine***

#### Project overview

**Fig. 14:**
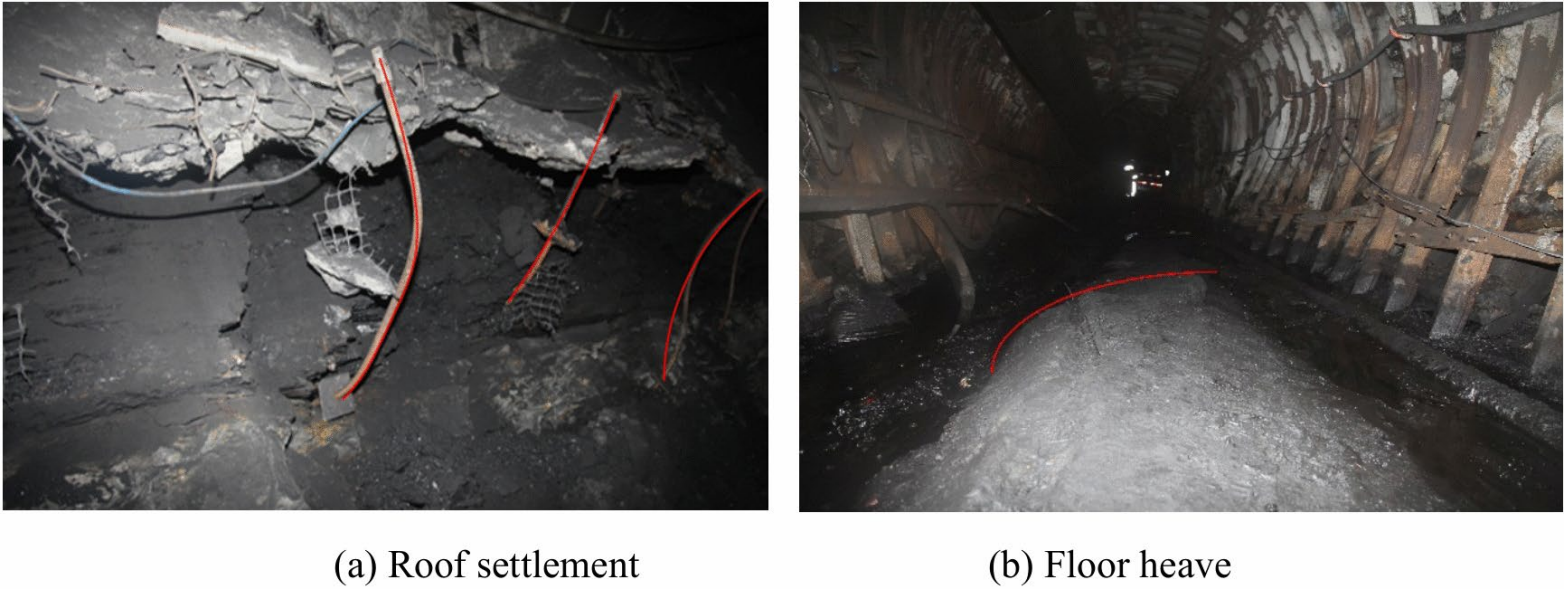
Deformation and failure of surrounding rock of roadway.

Shuangma mine is located about 60 km southeast of Lingwu City in the Ningxia. Currently, the main coal seams being mined are 4-2 and 4-3. The primary challenges faced are abnormal deformation and failure of the surrounding rock in the goaf of the I0104_3_02 working face caused by mining activities, as shown in Fig. 14. After identification, it belongs to large deformation failure.

#### "Multi-Level pressure yielding" control technology

##### Pressure yielding bolts + cable net support

**Fig. 15:**
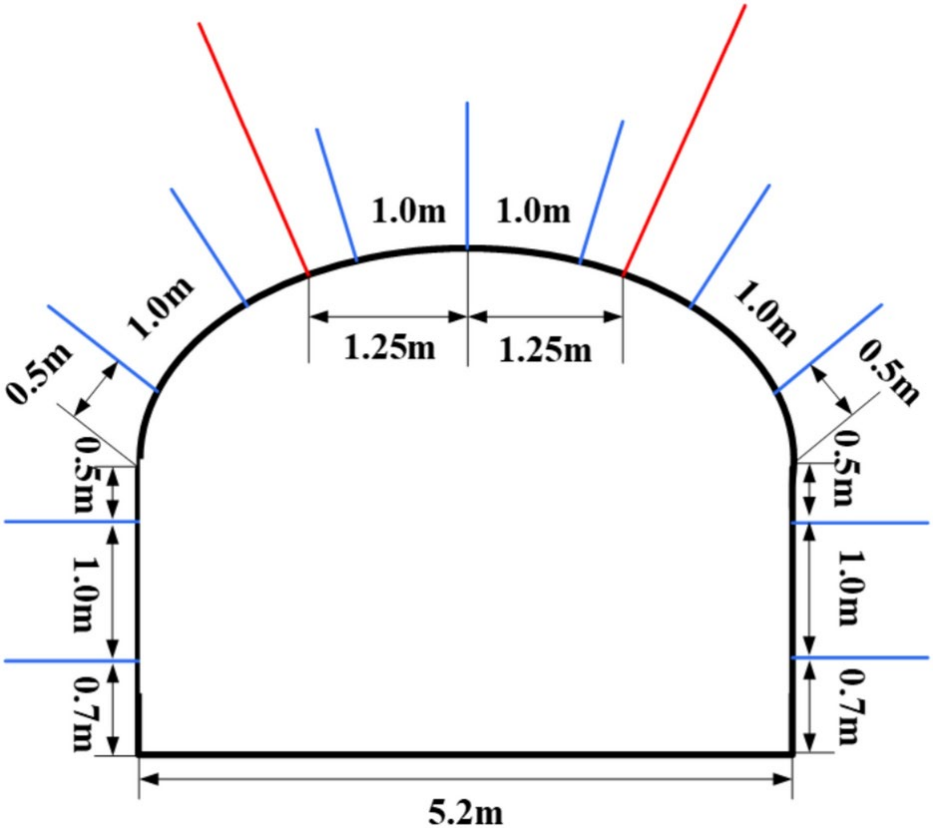
Support cross-sectional diagram of I0104_3_02 transportation roadway.

The top bolts are all pressure yielding bolts with a diameter of Φ22 × 2500 mm, each with 2 rolls of MSCK2370 type resin anchoring agent, and the bolt spacing is 2500 × 1300 mm. The tray specification is a 150 × 150 × 12 mm butterfly steel plates. The anchoring force of the bolts shall not be less than the standard value of the anchor bolt’s yield strength, which is 130 kN.^29^ The spacing between the side anchor bolts is 1000 × 1300 mm. For the I0104_3_02 working face transportation lane roof, anchor cables with a strength not lower than 356.43 kPa and a length not shorter than 4.06 m are selected, with a spacing of 1000 mm between the anchor cables and a row spacing of 2200 mm. Support cross-sectional diagram of I0104302 transportation roadway as shown in Fig. 15.

##### Shotcrete layer

**Fig. 16:**
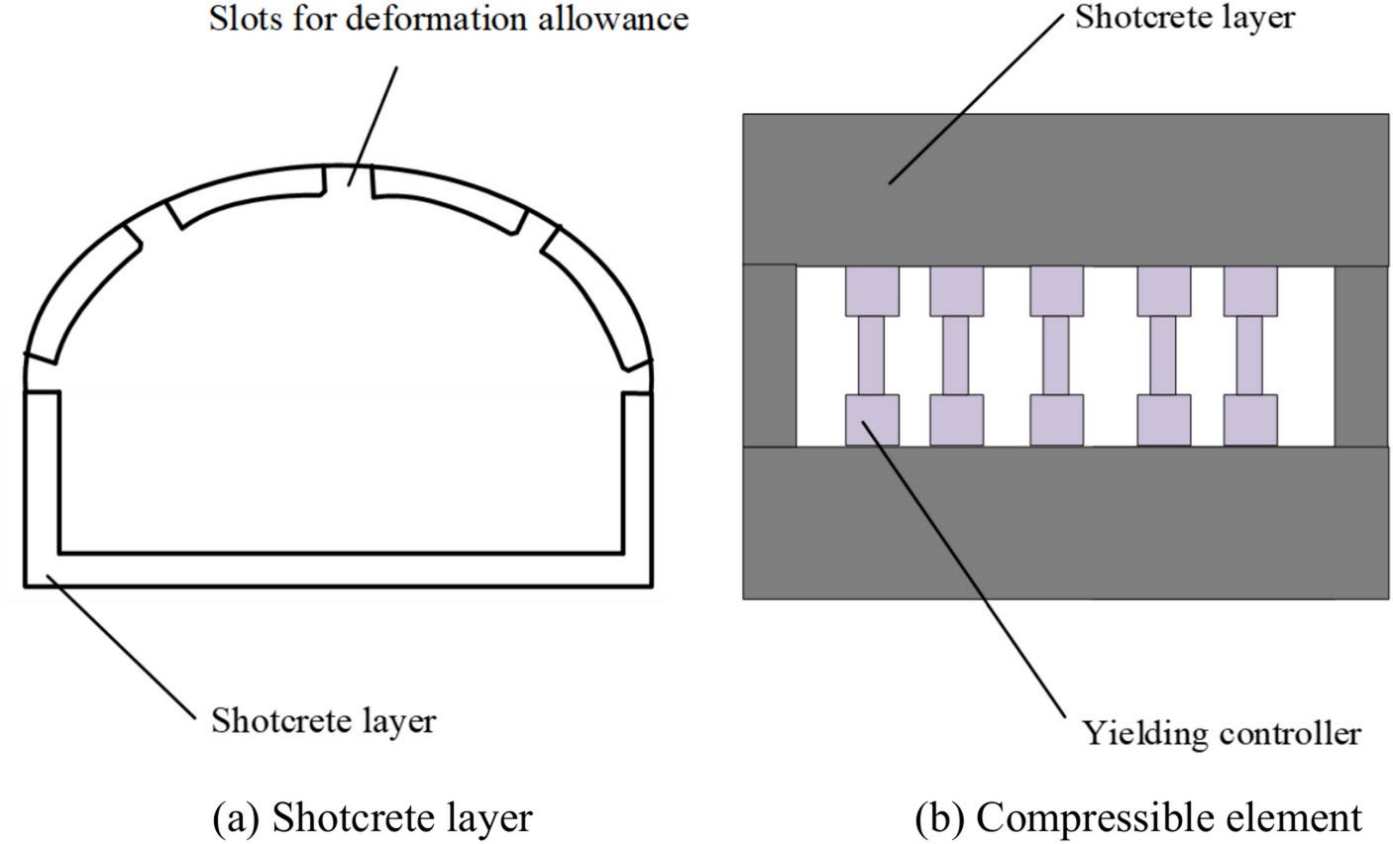
Shotcrete layer diagram.

Surface treatment is carried out using spraying materials,^30^ with a spraying thickness of 40–50 mm for the roof and both sides, a spraying strength of C20, and a spraying ratio of 1:2:2; the spraying material with a tensile strength of ≥ 3.0 MPa, an elongation rate of ≥ 20%, a bonding strength of ≥ 3.0 MPa, and a surface drying time of ≤ 60 min. Reserve deformation allowance slots during concrete spraying and install compression components in the slots. Shotcrete layer as shown in the Fig. 16.

#### Application effect analysis

**Fig. 17:**
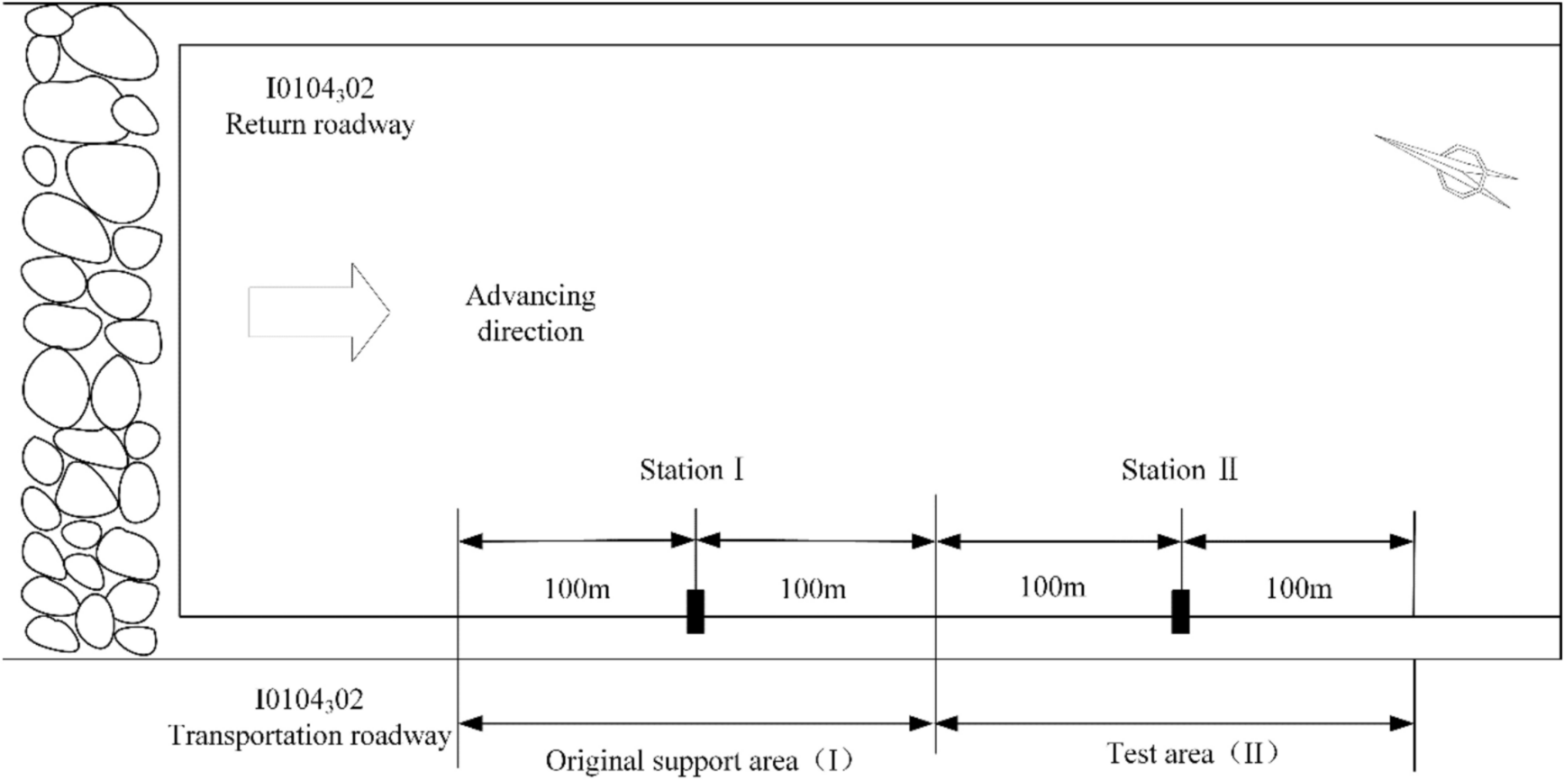
Schematic diagram of monitoring station layout.

This roadway surrounding rock monitoring technical plan mainly obtains on-site measured data such as roof separation and anchor/bolt force through monitoring methods such as anchor/bolt force gauges and roof separation instruments. A total of 4 observation stations are arranged in the I0104_3_02 working face transportation roadway, with 2 stations used to monitor the original support area (Area I) of the I0104_3_02 working face transportation roadway, and 2 stations used to monitor the "multi-level pressure yielding" control technology test area (Area II), as shown in Fig. 17.

##### Anchor bolts/Cables force monitoring

**Fig. 18:**
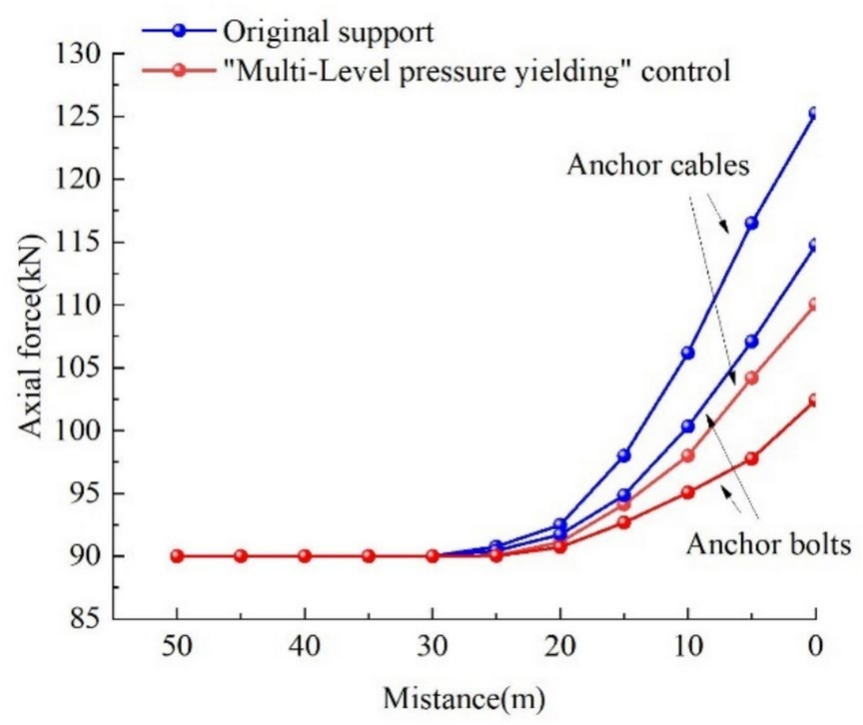
Force monitoring diagram of bolts/ cables.

In order to obtain the force situation of anchor bolts/cables in the I0104_3_02 transportation roadway and evaluate the support effect of the "multi-level pressure yielding" control technology,^31^ a anchor bolts/cables force gauge is used to monitor the roof of the I0104_3_02 transportation roadway. The monitoring results of the cable force gauge are shown in Fig. 18.

The monitoring results from the bolt/cable load cells show that under the original support conditions, when the working face advanced to 30 m away from the monitoring station, the axial forces of the bolts/cables began to increase due to mining-induced stress. The axial forces reached their maximum values when the working face advanced to the monitoring station, with the bolt axial force at 117 kN and the cable axial force at 133 kN. After implementing the "multi-level pressure yielding" integrated control strategy, the axial forces of the bolts/cables started to increase when the working face advanced to 27 m from the monitoring station under mining-induced stress and similarly peaked at the monitoring station. The bolt axial force was 103 kN, representing a reduction compared to the original support scheme, and the cable axial force was 110 kN, also showing a decrease. The monitoring results indicate effective support performance.

##### Roof separation monitoring

**Fig. 19:**
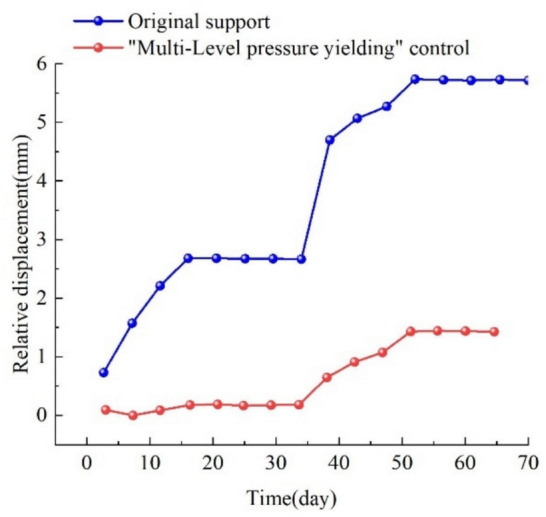
Roof separation monitoring.

In order to obtain the roof separation situation of the support optimization plan in the I0104_3_02 transportation roadway and evaluate the support effect of the "multi-level pressure yielding" control technology, a roof separation instrument is used to monitor the roof of the I0104_3_02 transportation roadway. The monitoring results of the roof separation instrument are shown in Fig. 19.

The monitoring results from the roof separation instruments reveal that during the mining of the I0104_3_02 working face, the relative displacement under the original support scheme continued to increase until stabilizing temporarily at 3 mm, indicating the development of fractures in the roadway roof due to concentrated stress from coal pillars. On day 35, the roof began to be affected by mining-induced stress, leading to a further increase in relative displacement until stabilization at 6 mm, demonstrating significant mining impact and progressive delamination of the roof. After implementing the "multi-level pressure yielding" integrated control, the relative displacement of the roadway roof showed minimal change during days 0–35, confirming effective control of roof delamination. On day 35, the relative displacement of roof delamination started to increase and stabilized at 1.5 mm, indicating the onset of mining-induced influence. Compared with the pre-control scenario, the relative displacement of roof delamination under the "unloading-support" strategy was reduced by 266%, with no significant delamination observed and intact shallow roof strata. The "multi-level pressure yielding" integrated control effectively protected the roof, demonstrating favorable support performance.

Based on the "multi-level pressure yielding" control design and on-site engineering practice, it is shown that:

1. After the "multi-level pressure yielding" control, the force on the anchor bolts/ cables in the I0104_3_02 transportation roadway was reduced by 12.0% and 17.3%, respectively, and the stability of the surrounding rock of the roadway was enhanced.
2. The relative displacement of the roof separation instrument in the I0104_3_02 transportation roadway was reduced by 266% compared to before the "multi-level pressure yielding" control, and the deformation and failure of the surrounding rock of the roadway were effectively controlled.

### Intake roadway of 6305 working face in Xinjulong mine

#### Project overview

**Fig. 20:**
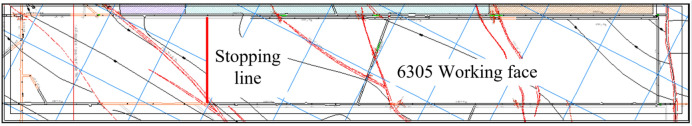
Plan view of 6305 working face.

Xinjulong Coal Mine is one of the mines with serious impact pressure hazards,^32^ and the 6305 working face is a demonstration working face for impact prevention in the mine. After identification, it has the risk of impact instability. Its designed strike length is 1390 m, and the dip length is 260.6–265.5 m. The coal layer is the 3rd coal layer, with a small variation in coal thickness, 8.1–10 m in thickness, an average coal thickness of 9.08 m, and a coal layer dip angle of 0°–16°, with an average of 8°. The plan view of the 6305 working face is shown in Fig. 20.

#### "Relief-Support" control technology

##### Blasting to break the roof

Dip blasting hole layout: 3 holes per group, spaced 7.5 m apart, with a hole diameter of 89 mm, drilled vertically down the side, with angles of 80°, 70°, and 50°, and hole depths of 51 m, 53 m, and 66 m, respectively, with charge weights of 75 kg, 78 kg, and 99 kg, respectively. Blast one group at a time.

Strike blasting hole layout: Arranged along the strike of the roadway, 2 strike blasting holes are arranged between two groups of dip blasting holes, with a hole spacing of 5m, an angle of 70°, and a hole depth of 50 m, with a charging amount of 75 kg.

##### Strengthened support

Between the roof steel straps, 29# U-shaped anchor cable beams (one beam with three cables) are added, with the anchor cable beam being 4500 mm long, the hole spacing being 1800 mm, and the row spacing being 1000 mm with double bearing plates. The anchor cables use Φ21.8 × 6300–10300 mm high-prestress steel strand, with an bond length of not less than 1500 mm and a pre-tightening force of not less than 200 kN. The side of the anchor cable beam is inclined 10°–30° towards the side.

**Fig. 21:**
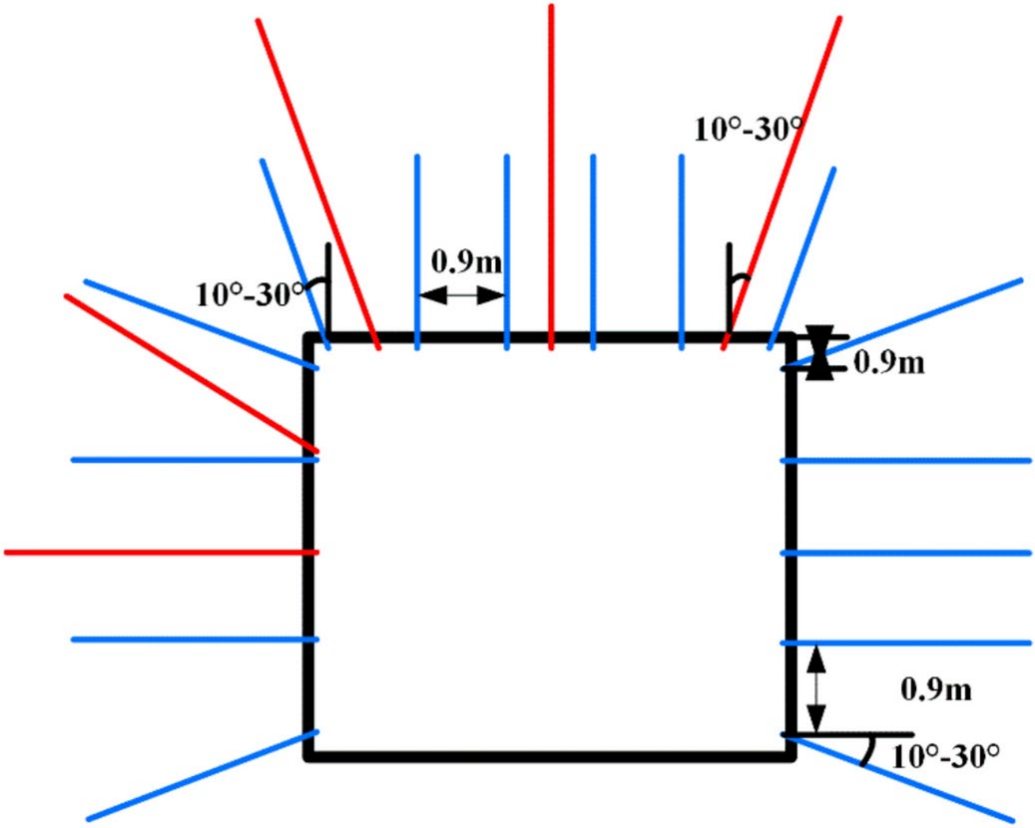
Permanent support cross-sectional diagram of 6305 intake airway.

The west side is supplemented with 2 U-type anchor cable beams (one beam with two cables), with a length of 2600 mm, hole spacing of 2000 mm, and row spacing of 2000 × 2000 mm. The anchor cables use Φ21.8 mm × 8300 mm steel strand anchor cables, with the lower hole position drilled vertically down the side. Permanent support cross-sectional diagram of 6305 intake airway as shown in Fig. 21.

#### Application effect analysis

**Fig. 22:**
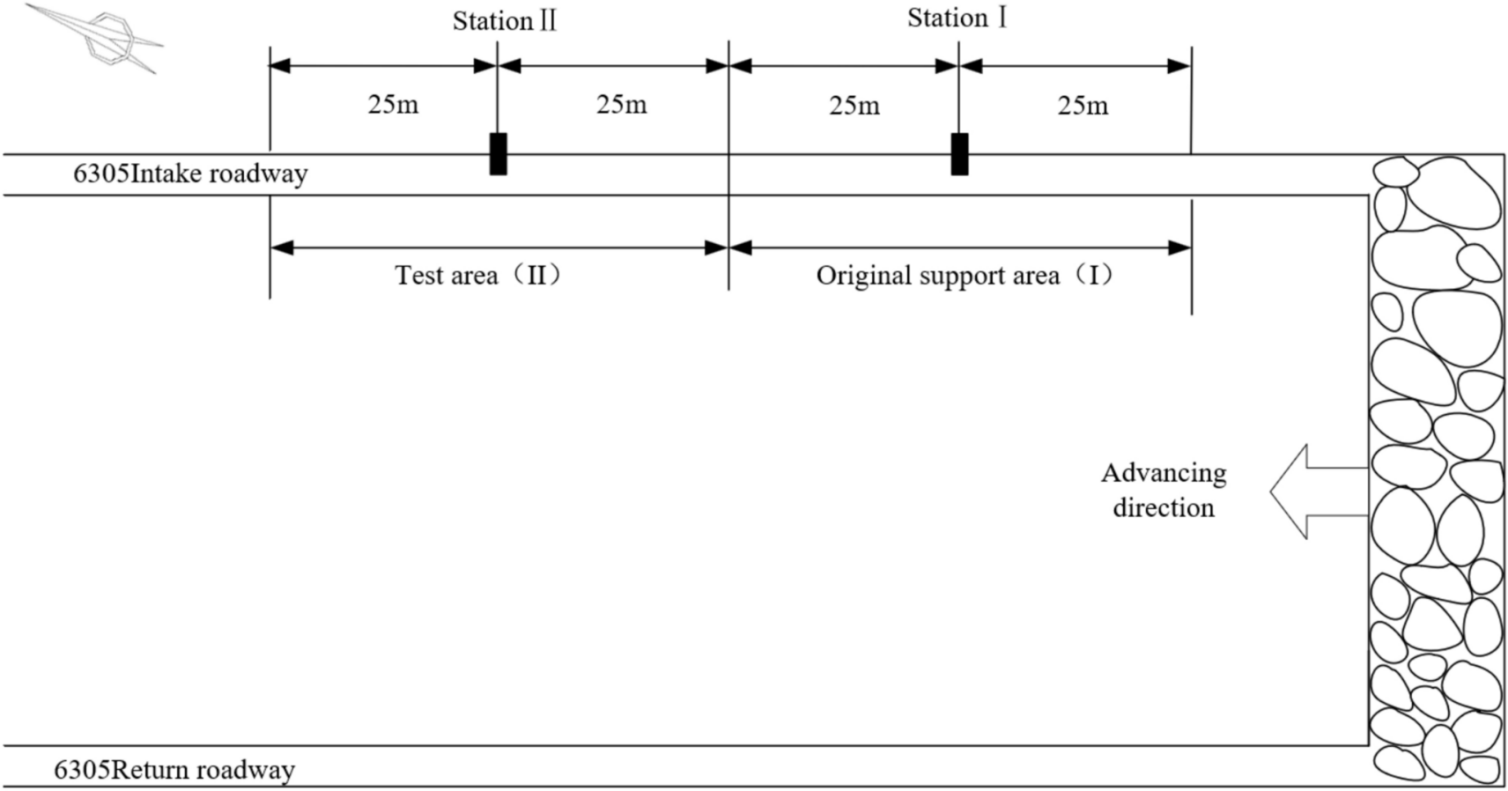
Schematic diagram of monitoring station layout.

This roadway surrounding rock monitoring technical plan mainly obtains on-site measured data of surrounding rock damage and microseismic events through monitoring methods such as microseismic monitoring and drilling debris method.^33^ A total of 2 observation stations are arranged in the 6305 working face intake roadway, with 1 station used to monitor the original support area (Area I) of the 6305 working face intake roadway, and 1 station used to monitor the "Relief-Support" control technology test area (Area II), as shown in Fig. 22.

##### Microseismic analysis

By counting the number and energy size of microseismic events in the 6305 working face intake roadway, the number and proportion of microseismic events with high energy before and after the "Relief-Support" control are obtained.

**Fig. 23:**
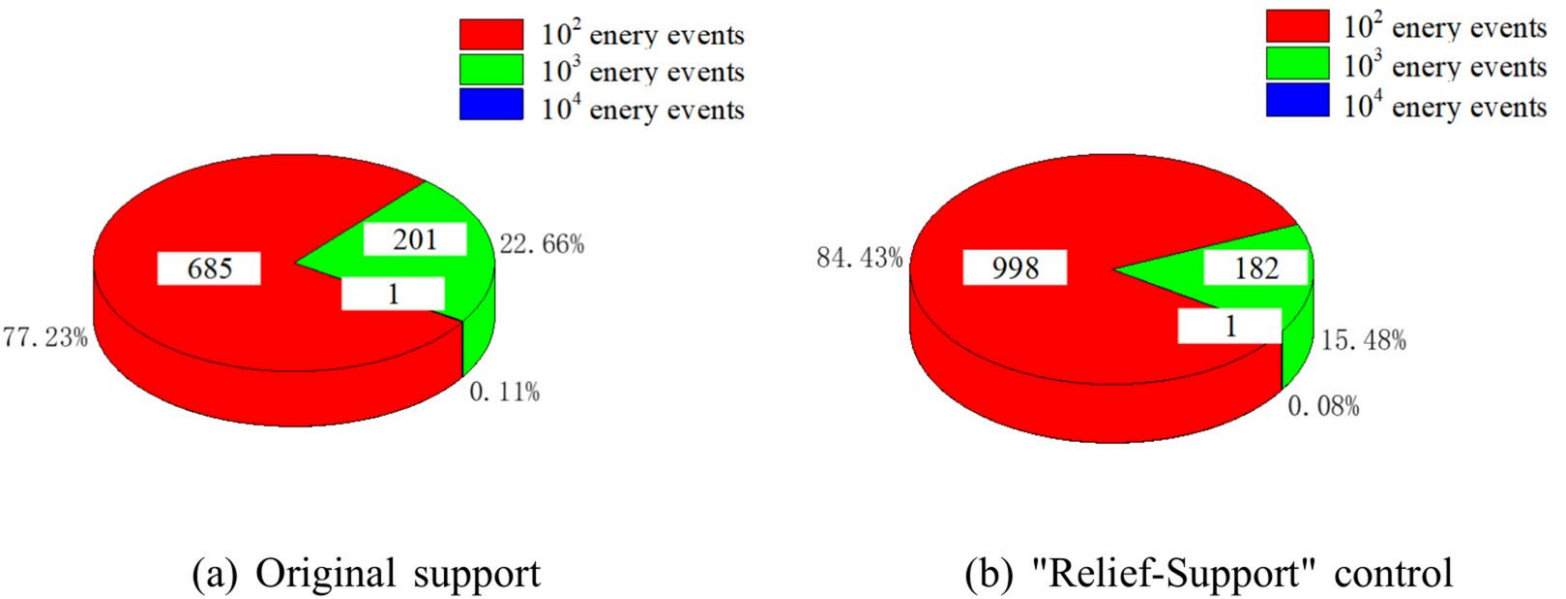
Comparison of high energy microseismic events (**a**) Original support (**b**) "Relief-Support" control.

As shown in Fig. 23, before the "Relief-Support" control, microseismic events with energy greater than 10^3^J account for 22.77% of the total number of microseismic events, and events with energy exceeding 4 × 10^4^J have occurred. Compared with before the "Relief-Support" control, the microseismic energy fluctuation curve is more uneven, and high energy events occur more frequently. After the "Relief-Support" control, microseismic events greater than 10^3^J account for 15.56% of the total microseismic events, a decrease of 7.21%. From the "Relief-Support" control point onwards, the overall microseismic energy fluctuation is more stable, and the frequency of high-energy events is reduced.

##### Drill cuttings analysis

By organizing the results of the drill cuttings monitoring in the 6305 working face intake roadway, analyses were conducted at locations 10 m and 20 m ahead of the measuring station, and 10 m and 20 m behind the measuring station, before and after the "Relief-Support" control. The coal powder quantity before and after the "Relief-Support" control is shown in Tables 2 and 3.

**Table 2:** Drill cuttings monitoring before "Relief-Support" control.

Location	Dynamic effects	Coal quantity (kg)														
		1	2	3	4	5	6	7	8	9	10	11	12	13	14	15
Before station I 20 m	Not	1.0	2.0	1.9	2.0	1.6	2.4	2.9	2.0	3.4	1.5	2.6	1.7	3.7	2.4	2.6
Before station I 10 m	Not	1.2	1.4	2.0	1.5	2.5	1.5	2.7	2.1	2.5	4.3	2.4	1.5	2.0	1.7	3.2
After station I 10 m	Not	1.2	1.8	1.9	2.0	2.6	2.5	3.1	3.7	3.5	2.3	2.5	2.6	2.6	3.0	3.0
After station I 20 m	Not	1.7	1.6	2.0	2.4	2.1	2.9	1.7	2.6	2.9	2.0	2.4	2.0	3.7	1.9	1.1

**Table 3:** Drill cuttings monitoring after "Relief-Support" control.

Location	Dynamic effects	Coal quantity (kg)														
		1	2	3	4	5	6	7	8	9	10	11	12	13	14	15
Before station II 20 m	Not	1	1.5	1.7	2	1.9	2.1	2	2.5	2.3	2.4	2.1	2.8	2.6	2.5	3.1
Before station II 10 m	Not	1.4	1.8	1.6	1.8	2	1.9	2.3	1.9	2.4	2.3	2	2.4	2.3	2	3.5
After station II 10 m	Not	1.2	1.7	1.9	2	2.3	2.1	2.5	2	2.7	4	3.2	2.3	3.4	3.3	2.5
After station II 20 m	Not	1.1	1.5	1.7	1.7	1.2	1.4	2.1	1.2	2	2.3	2.1	1.9	2.3	1.3	1.7

**Fig. 24:**
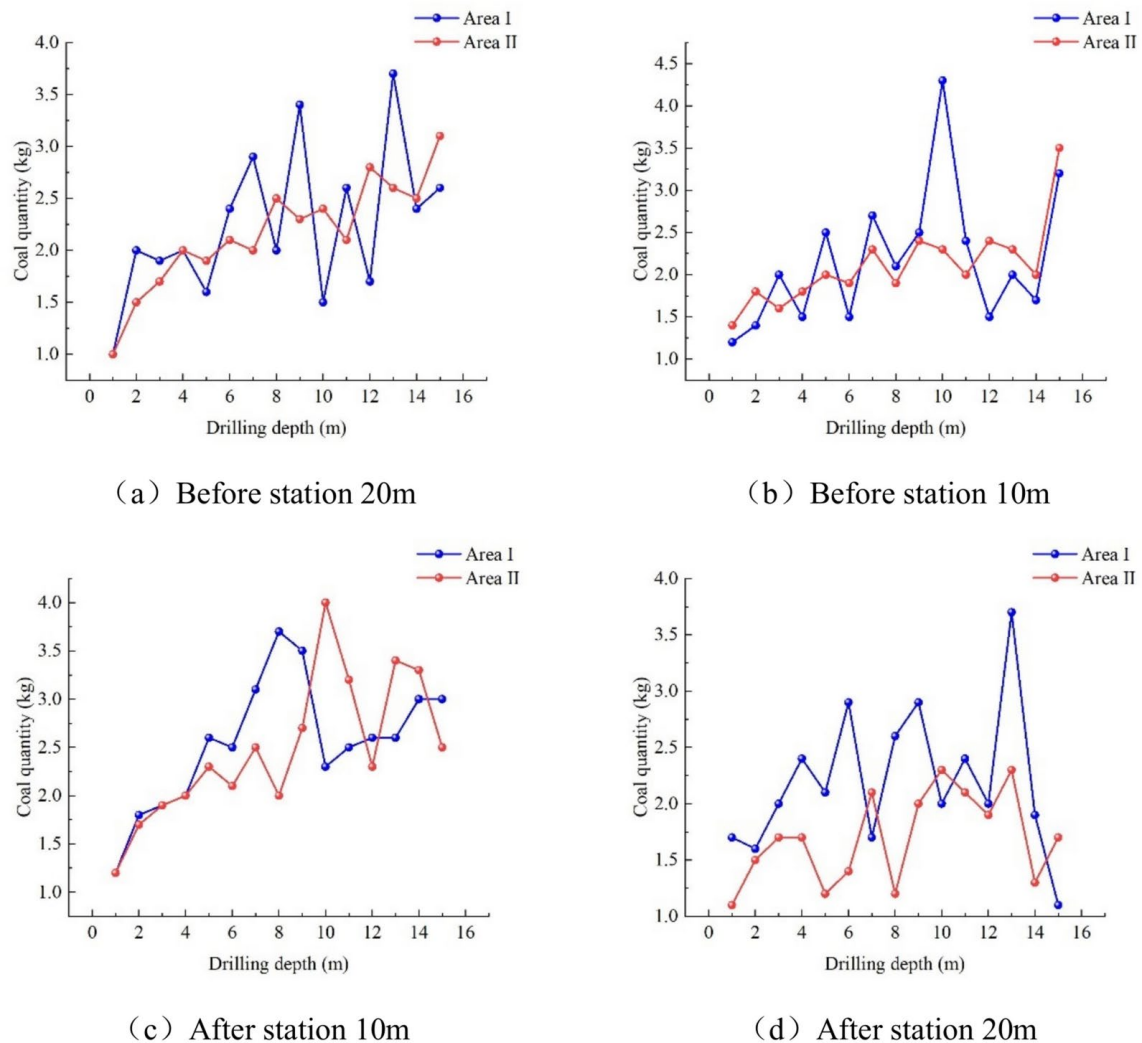
Comparison curve of coal powder quantity.

As shown in Fig. 24, after the implementation of the "Relief-Support" control measures, the amount of coal cuttings has decreased compared to before the "Relief-Support" control. The drilling powder rate index, which indicates the risk of impact danger at the workplace, has also significantly decreased, indicating a clear reduction in the risk of impact danger in this area.^34,35^

Based on the "Relief-Support" control design and on-site engineering practice, it is shown that:

(1) After the "Relief-Support" control, the proportion of microseismic events greater than 10^3^ J decreased by 7.21%, and the overall microseismic energy fluctuation is more stable, with a reduced frequency of high-energy events.

(2) After the "Relief-Support" control, the dynamic effect of the workplace’s impact danger showed no change, but the drilling powder rate index, which indicates the risk of impact danger, decreased, indicating a clear reduction in the risk of impact danger in this area.

## Conclusion

1. Based on numerical simulation, the advance influence range of mining roadway is approximately 30 m. The energy influence range and value at both sides of the roadway gradually increase with the mining of the working face, reaching the limit at 10 m ahead of monitoring line. Then it spreads from both sides towards the roof and floor and reaches its maximum in face end, forming a butterfly-shaped dissipation area and a trapezoidal elastic area. The surrounding rock of the roadway is divided into energy consumption zone, energy supply zone, and unaffected zone based on the range
2. From the aspects of energy level and energy release rate, a method for identifying the types of surrounding rock deformation and failure is proposed. When the energy level identification coefficient *K* ≥ 1 and the energy rate identification coefficient *V* ≥ 1, the surrounding rock of the roadway undergoes impact instability; when the energy rate identification coefficient *V* < 1, the surrounding rock of the roadway undergoes large deformation failure.
3. A "Multi-Level pressure yielding" control technology for surrounding rock of roadway at risk of large deformation was proposed and applied in Shuangma Mine. On-site monitoring results show that after adopting the control technology, the force on anchor bolts/cables was reduced by 12.0% and 17.3%, respectively, and the relative displacement of the roof separation instrument was reduced by 266%. The deformation and failure of surrounding rock were effectively controlled.
4. An "Relief-Support" control technology for surrounding rock of roadway at risk of impact instability was proposed and applied in Xinjulong. On-site monitoring results show that after adopting the control technology, the proportion of large energy microseismic events decreased by 7.21%, and the drilling powder rate index decreased, reducing the risk of impact instability in the roadway.

## Data Availability

The original contributions presented in the study are included in the article material. Further inquiries can be directed to the corresponding author. The data used to support the findings of this research are included within the paper.
